# Aerosol Optical Retrieval and Surface Reflectance from Airborne Remote Sensing Data over Land

**DOI:** 10.3390/s100706421

**Published:** 2010-06-30

**Authors:** Cristiana Bassani, Rosa Maria Cavalli, Stefano Pignatti

**Affiliations:** 1 Institute for Atmospheric Pollution (IIA), Italian National Research Council (CNR), Division Airborne Laboratory for Environmental Research (LARA), Research Area of Roma-2 in Tor Vergata, Via Fosso del Cavaliere 100, 00133 Rome, Italy; E-Mail: cavalli@lara.rm.cnr.it (R.M.C.); 2 Institute of Methodologies for Environmental Analysis (IMAA), Italian National Research Council (CNR), C.da S. Loja, 85050 Tito (Potenza), Italy; E-Mail: pignatti@lara.rm.cnr.it (S.P.)

**Keywords:** atmospheric radiative transfer, aerosol optical thickness, atmospheric correction, hyperspectral remote sensing, reflectance, remote sensing

## Abstract

Quantitative analysis of atmospheric optical properties and surface reflectance can be performed by applying radiative transfer theory in the Atmosphere-Earth coupled system, for the atmospheric correction of hyperspectral remote sensing data. This paper describes a new physically-based algorithm to retrieve the aerosol optical thickness at 550*nm* (*τ*_550_) and the surface reflectance (*ρ*) from airborne acquired data in the atmospheric window of the Visible and Near-Infrared (VNIR) range. The algorithm is realized in two modules. Module A retrieves *τ*_550_ with a minimization algorithm, then Module B retrieves the surface reflectance *ρ* for each pixel of the image. The method was tested on five remote sensing images acquired by an airborne sensor under different geometric conditions to evaluate the reliability of the method. The results, *τ*_550_ and *ρ*, retrieved from each image were validated with field data contemporaneously acquired by a sun-sky radiometer and a spectroradiometer, respectively. Good correlation index, *r*, and low root mean square deviations, *RMSD*, were obtained for the *τ*_550_ retrieved by Module A (*r*^2^ = 0.75, *RMSD* = 0.08) and the *ρ* retrieved by Module B (*r*^2^ ≤ 0.9, *RMSD* ≤ 0.003). Overall, the results are encouraging, indicating that the method is reliable for optical atmospheric studies and the atmospheric correction of airborne hyperspectral images. The method does not require additional at-ground measurements about at-ground reflectance of the reference pixel and aerosol optical thickness.

## 1. Introduction

The hyperspectral remote sensing data collected by sensors on-board satellite and aircraft platforms meet the requirements of imaging spectrometry by reproducing the reflectance or emittance spectrum of an image pixel with a fine-spectral resolution [1]. In the last few years, hyperspectral data in the 400 − 2, 500 nm spectral domain have been driving physical approaches for the quantitative analysis of land surface properties in fields of research such as geology, agriculture and urban studies [2–4]. The recognition of spectral features of the surface reflectance from at-sensor radiance issued to the definition of an accurate Atmospheric Correction (AC) pre-processing [1,5]. The AC algorithms for hyperspectral data acquired over land are based on an empirical approach [1] or on the physical model of the radiative field in the Atmosphere-Earth coupled system [6,7]. In the latter case, the description of the radiative field during the aircraft or satellite overpass also allows the retrieval of atmospheric parameters such as the aerosol optical thickness at 550 nm, *τ*_550_.

The most common AC based on the empirical approaches, which are devoted to retrieving only the surface reflectance without knowledge of the radiative field is the empirical line [8]. This method requires field reflectance measurements of the brightest and darkest pixels of the image. The principal limitations of the empirical approach are related to the choice of reference reflectance. The results of the AC applied to the hyperspectral data can highlight uncorrected spectral behavior because the absorption features of the reference reflectance are not completely spectrally flat, and they can be affected by different atmospheric attenuation, thus showing unrealistic features in the spectral reflectance of the pixel.

To overcome these limitations, physically-based approaches are used. These approaches provide “accurate and mathematically justified solutions” to the beam propagation in the Atmosphere-Earth coupled system [9]. The physically-based AC algorithm simulates the atmospheric effects on the at-sensor signal due to the absorption and scattering processes by using the theoretical model of the radiative field as a function of the constituents’ properties. In particular, these properties are (*i*) the columnar content of the absorber gas inside the absorption band and (*ii*) the optical properties of the aerosol along the entire Visible and Near Infra-Red (VNIR) spectral domain, affecting principally the atmospheric transmittance in the Visible domain. The retrieval of these atmospheric properties leads to the removal of the real atmospheric contributions from the at-sensor signal. In this way, by solving the inverse problem, it is possible to determine the radiative quantities if the at-sensor signal is known.

The radiative transfer in the atmosphere is simulated by radiative transfer codes such as the Moderate Resolution Transmittance (MODTRAN) [10] and the Second Simulation of a Satellite Signal in the Solar Spectrum (6S) [11]. The MODTRAN code is usually used in software packages developed for atmospheric corrections such as Fast Line-of-sight Atmospheric Analysis of Spectral Hypercubes (FLAASH), Atmospheric CORrection Now (ACORN) and ATmospheric REMoval algorithm (ATREM), as described in [12]. These packages are built to perform the atmospheric correction of remote sensing data and are used to estimate the columnar content of water vapor from the at-sensor signal by using differential absorption techniques such as the split-window applied to channels falling into the absorption bands and into neighboring atmospheric windows. The aerosol retrieval from the hyperspectral sensor is performed in the FLAASH package by an automated band-ratio method applied to specific channels of dark pixels [13] without taking advantage of all of the information contained in the hyperspectral imagery, [14]. ACORN uses a proprietary method for visibility spectral shape matching between 400 nm and 1, 000 nm with reference tables. With regards to the ATREM package, the aerosol retrieval from the hyperspectral sensor is limited to the aerosol effects by solving the direct problem which means that the aerosol properties, such as the aerosol optical thickness, need to be selected by the user.

The 6S radiative transfer code is an open-source code with a reasonable computational time with computing facilities to implement an atmospheric correction algorithm for specific sensors. The last generation, vector version, 6Sv1.1 code [9], significantly improves the accuracy of the remote sensing results, such as for the MODIS (Moderate Resolution Imaging Spectroradiometer) products [15]. The code is free and downloadable from *http://modis-sr.ltdri.org/6S_code/index.html*.

The physically-based approach is able to retrieve the aerosol optical thickness from the at-sensor radiance in the atmospheric window of the 400 − 2, 500 nm spectral domain. Thus, the aerosol optical thickness has become a key atmospheric parameter to study the at-sensor signal of sensors working in the VNIR spectral domain [16]. Moreover, in the last year the correlation between the aerosol optical thickness retrieved from optical remote sensing data and the Particulate Matter (PM) has been studied [17,18] to evaluate the relevant representation of aerosol optical properties in monitoring the atmospheric pollution in specific areas [19].

At present, few case studies on aerosol optical retrieval from hyperspectral data for modeling the scattering effects have been reported [1]. The most recent method was applied for the first time to the data acquired by the hyperspectral Compact High Resolution Imaging Spectrometer (CHRIS) sensor on board the PRoject for On-Board Autonomy (PROBA) satellite [20] and by the multispectral MEdium Resolution Imaging Spectrometer instrument (MERIS) sensor on board the ENVIronmental SATellite (ENVISAT) [21]. The method, if applied to multispectral data, does not show good performance for retrieving the optical properties of the atmosphere [21]. For airborne hyperspectral remote sensing data, the method has been applied to Compact Airborne Spectrographic Imager (CASI) data [5].

In this work, a new approach to solve the inverse problem (aerosol optical retrieval) by using weight contribution of each sensor channel (free parameters) falling into the atmospheric window of the VNIR spectral domain, is presented. The method takes advantage of the large amount of spectral information provided by the contiguous channels of a sensor with high spectral resolution to better identify the spectral atmospheric radiative effects of the aerosol scattering on the at-sensor radiance without any at-ground measurements [1] and working under the usual Lambertian assumption [5]. In addition, this method is able to retrieve the aerosol optical thickness without any empirical relationship between channels’ surface reflectance which is a usual assumption of the aerosol retrieval algorithms developed for multispectral remote sensing data. For example, the MODIS algorithm for aerosol optical thickness retrieval assumes the correlation between the surface reflectance of the channels in the visible and near infrared (2.1*μm*) spectral domains [13].

Nevertheless, the presented method works with channels inside the atmospheric windows of VNIR spectral domain. Thus, pressure, ozone and water vapor (retrievable by using sensor channels falling into absorption bands of the water vapor and the oxygen) cannot be derived by this method; consequently their profiles are assumed by using atmospheric standard models, such as midlatitude summer and midlatitude winter.

The presented method is composed of two modules explained in Section 2. The first Module (Module A) is dedicated to the minimization algorithm that retrieves the aerosol optical thickness, *τ*_550_, from the airborne data (inverse problem) by an iterative process, Section 2.1. The inability of the method to retrieve information about the aerosol model requires to take the aerosol optical and microphysical parameters, basically single scattering albedo and phase function, from existing models implemented in the atmospheric radiative transfer code. To gauge the Module A’s performance, its results were compared with the at-ground measurements of *τ*_550_ acquired using a sun-sky radiometer. The retrieved aerosol optical thickness was further used to accurately simulate the scattering effects on the atmospheric radiative field (direct problem).

Module B for the AC algorithm for airborne data starting from the retrieved *τ*_550_ is explained in Section 2.2. The pixel-based approach to calculating the reflectance value, *ρ*, of each pixel of the image is based on the analytical expression of the reflectance viewed by an airborne sensor [6]. The final step of Module B removes the blurring image due to the radiation scattered from the environmental target and reaching the sensor, namely, the adjacency effect.

The method was tested on remote sensing data that were acquired on July 26, 2001, and July 20, 2002, by the MIVIS (Multispectral Infrared and Visible Imaging Spectrometer) airborne sensor [22], which records 102 spectral channels in the visible, near-infrared, short-infrared and thermal spectral domains, [23–28], as described in Section 3.

## 2. Methods

The novel approach of the method is a theory-based radiative transfer strategy to retrieve the aerosol optical thickness at 550 nm, *τ*_550_, and consequently the surface reflectance for each pixel, *ρ*, by the AC of the remote sensing data. The methodology follows the 6S analytical expression of the target reflectance viewed at *z* altitude by an airborne sensor [11], *ρ^sim^*, decoupling gaseous absorption from the scattering processes for a Lambertian surface characterized from the isotropy of the Bi-directional Reflectance Distribution Function (BRDF) from the illumination and viewing angles:

(1) $$\rho^{\mathrm{sim}}\;(\mu_{s},\;\mu_{v},\;\phi ,\;z)\;=\;T^{\mathrm{gas}}\;(\mu_{s},\;\mu_{v},z)\;\left[\rho^{\mathrm{atm}}\;(z)\;+\;\frac{T^{↓}\;(\mu_{s})T^{↑}\;(\mu_{v},\;z)\rho^{s}}{1\;-\;S\rho^{s}}\right]$$

Here, *ρ^sim^* is the at-sensor reflectance that is *ρ^sim^* = *πL^sim^/μ_s_E_s_*, where *L^sim^* is the at-sensor radiance, *E_s_* is the solar flux at the top of the atmosphere and *μ_s_* is the cosine of the solar zenith angle. The terms on the right side of Equation 1 are the surface reflectance (*ρ^s^*) and the atmospheric quantities joined to the radiative field in the coupled system. The at-sensor radiance, like all of the radiative quantities of the equation, depends on the radiation line-sight expressed by the angular variables: the cosine of the sun (*θ_s_*) and view (*θ_υ_*) zenith angles, *μ_s,υ_* = cos *θ_s,υ_*, and the relative azimuth between the solar and view azimuths, *ϕ* = *ϕ_s_* *−* *ϕ_υ_*. The angular dependencies of the radiative quantities will be omitted in the remainder of the paper for the sake of simplicity. The radiative quantities of Equation 1 are the intrinsic reflectance of the molecule and aerosol layer, *ρ^atm^*, called path reflectance; the spherical albedo, *S*; and the components of the flux transmission. The *T^gas^*(*μ_s_*, *μ_υ_*, *z*) is the gaseous transmittance, whereas the *T^↓^* = *e^−τ/μ_s_^* + *t_d_*(*μ_s_*) and *T^↑^* = *e*^*−τ*(*z*)/μ_υ_^ + *t_d_*(*μ_υ_*) are the summed direct and diffuse components, respectively, of the total transmittance for the illumination (descending) and view (ascending) directions. The *τ* is the total optical thickness and *τ*(*z*) is the optical thickness of the layer under the aircraft.

All of the mentioned spectral radiative quantities of Equation 1 are computed in the entire spectral domain of 400 − 2, 500 nm sampled every 2.5 nm. Thus, defining *g^j^*(λ*_l_*) to represent the set (*T^gas^*, *ρ^atm^*, *E^sun^*, *T^↓^*, *T^↑^* and *S*), *g^j^*(λ*_l_*) corresponds to the *j^th^* radiative quantity values that are continuous within the discrete-spectral domain λ*_l_* ∈ {λ*_min_* = 400*nm*, …, λ*_max_* = 2, 500*nm*} and are sampled every 2.5*nm*. To solve the equation for the *i^th^* sensor channel, the *g^j^*(λ*_l_*) have to be convolved to the spectral response function, *f_i_*(λ*_l_*) sampled every 2.5 nm, for the *i^th^* channel of the desired remote sensing sensor. The spectral convolution is given by the following equation:

(2) $$g_{i}^{j}\;=\;\frac{\sum_{\lambda_{l}=\lambda_{\min}}^{\lambda_{\max}}g^{j}\;(\lambda_{l})f_{i}(\lambda_{l})}{\sum_{\lambda_{l}=\lambda_{\min}}^{\lambda_{\max}}f_{i}(\lambda_{l})}$$

After the convolution, the *j^th^* radiative variable retrieved for the *i^th^* sensor channel, $g_{i}^{j}$, is ready to be introduced into Equation 1.

$\rho_{i}^{s}$ is the unknown variable, one for each sensor channel, of Equation 1. To solve Equation 1, all of the radiative quantities are simulated by the 6S radiative transfer code, whereas $\rho_{i}^{s}$ is defined by the free parameters that are retrievable by means of Module A of the presented method.

### 2.1. Retrieval of the Aerosol Optical Thickness, τ_550_ - Module A

The retrieval of the atmospheric parameters starting from the at-sensor radiance is an inverse problem. The most relevant parameter in the solar spectral domain is the aerosol optical thickness at 550 nm, *τ*_550_. This parameter is retrieved by the algorithm to minimize the following cost function:

(3) $$\delta^{2}\;=\;\sum_{p=1}^{n\geq 2}\sum_{i=1}^{\mathrm{chn}}\frac{1}{\lambda_{i}^{2}}{\left[\rho_{p,i}^{\mathrm{meas}}\;-\;\rho_{p,i}^{\mathrm{sim}}\right]}^{2}$$

This represents the difference between the at-sensor radiance ( $L_{i}^{\mathrm{meas}}$) for the *i^th^* channel as measured by the sensor, expressed in terms of equivalent reflectance $\rho_{i}^{\mathrm{meas}}\;=\;\pi L_{i}^{\mathrm{meas}}/\mu_{s}E_{s}$, and that obtained from Equation 1, $\rho_{i}^{\mathrm{sim}}$, where all the radiative quantities, $g_{i}^{j}$, are simulated with 6S code and convolved to the spectral response function of the *i^th^* sensor channel using Equation 2.

The λ*_i_* is the center wavelength of the *i^th^* channel in *μm*, and it drives the minimization toward the spectral region in the domain of 400 − 2, 500 nm where the effects of the aerosol attenuation have more weight, that is, in the Visible spectral range. The double sum is defined over all of the sensor channels falling into the atmospheric window of the VNIR spectral domain, *i*, and the spectrally homogeneous pixels, *n*, belonging to the study area chosen to test the method, where *n* ≥ 2.

The minimization of the cost function, *δ*^2^, performed on the *n* pixels of the study area also allows the evaluation (for each sensor channel) of the $\rho_{i}^{s}$ of Equation 1 by using one variable value, for each channel. This characteristic, a new aspect of the method explained in [20], takes advantage from the large number of channels offered by the hyperspectral dataset to better distinguish spectrally different atmospheric effects on the at-sensor radiance.

To solve the Equation 3 with respect to unknown parameters *τ*_550_ and all the $\rho_{i}^{s}$ values, we relied on the Powell method [29,30]. The Powell method is highly robust and has been widely used in the literature for solving inverse problems in remote sensing [21,31–33].

The Powell method requires initialization of the unknown parameters $\rho_{i}^{s}$ for proceeding with iterations. To this purpose they were computed as $\rho_{i}^{s}\;=\;c_{i}\rho_{i}^{\mathrm{ref}}$, where $\rho_{i}^{\mathrm{ref}}$ are the reference reflectance spectrum convolved to the sensor channels and selected from the predefined spectral library. The parameter *c_i_* has been set to *c_i_* = 0.01 for all wavelengths. This value has been chosen from numerical experiments since it guarantees convergence to the global minimum of Equation 3. They do not represent the real abundance of $\rho_{i}^{\mathrm{ref}}$, but are only used for the startup of the Powell method (Module A). The validation of Module A will be performed on the values of the aerosol optical thickness retrieved from the module and measured at-ground by the Cimel sun-sky radiometer.

### 2.2. Retrieval of the at-Ground Pixel Reflectance, ρ - Module B

The aerosol optical thickness (*τ*_550_), retrieved from Module A, allows an accurate simulation of the radiative quantities, $g_{i}^{j}$, in the atmospheric window of the VNIR spectral domain by using the 6S radiative transfer code. The $g_{i}^{j}$, introduced in the Equation 1, solve the equation for the unknown variable, *ρ^s^*, on each pixel of the image.

*τ*_550_ is the atmospheric input parameter required by the 6S code to accurately simulate the radiative field during the aircraft overpass (direct problem), whereas, the geometric parameters of the acquisition are required for input into the 6S code to simulate the atmospheric radiative effects on the radiation line-sight. Regarding the solar geometry, the code inputs are the sun zenith (*θ_s_*) and azimuth (*ϕ_s_*) angles, whereas, the viewing geometry is defined by the scan-angle of each pixel seen by the sensor. The scan-angle variable is represented in the 6S code by the input, *i.e.*, the view zenith (*θ_υ_*) and azimuth (*ϕ_υ_*) angles calculated for the pixel seen by the sensor.

The simulation of the radiative quantities, performed by a pixel-by-pixel method over a scan line, requires a considerable computational processing time (60*sec/pixel*). Consequently, the 6s code runs just for the viewing geometry of six pixels, equidistant along a scan line. The radiative quantities simulated on the six pixels are interpolated along all of the scan line pixels. Thus, the Equation 1 is analytically inverted to determine the $\rho_{i}^{s}$ for each pixel by using the values of the retrieved $g_{i}^{j}$. The result is the image expressed in reflectance, where the value of each sensor channel, $\rho_{i}^{s}$, still includes the environmental contribution to the actual reflectance, *ρ_i_*. Therefore, the radiative contribution to the surface reflectance is removed from each pixel by using the following equation, derived from the equation for adjacency effect correction presented in [6]:

(4) $$\rho_{i}\;=\;\rho_{i}^{s}\;+\;\frac{t_{d}{\left(\mu_{v}\right)}_{i}}{e_{i}^{-\tau /\mu_{v}}}[\rho_{i}^{s}\;-\;<\rho_{i}^{s}>]$$

The 6S code permits the decoupling of the direct, *e^−τ/μ_υ_^*, and the diffuse, *t_d_*(*μ_υ_*), components of the transmittance along the pixel-sensor path. < $\rho_{i}^{s}$ > is the average reflectance of the pixels adjacent to the observed pixel calculated for the *i^th^* sensor channel, taking a square of 200 × 200 pixels around the observed pixel. The empirical formula leads to solving the AC using the simplest approach, taking into account the adjacency effect during the AC of the airborne sensor.

## 3. Experimental Section

### 3.1. Study Area and Data

The study area was selected following the requirements needed to evaluate the performance of the proposed method: (*i*) the pixels of the study area must be spectrally homogeneous and have isotropic reflectance; (*ii*) the chosen area has to be recorded by a sensor with different geometric conditions, which means different viewing geometries (view zenith and azimuth angles) and different times (solar zenith and azimuth angles); (*iii*) the at-ground measurements in the same area have to be collected for validation purposes.

**Figure 1.**
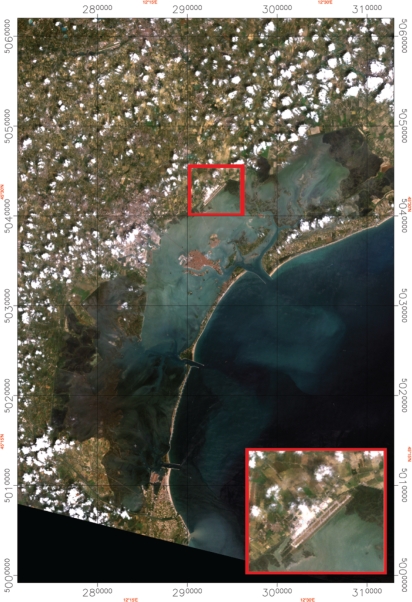
The study area meets the requirements of the method: the Venice airport.

Contemporaneously with the airborne campaigns, measurements of *τ*_550_ and at-ground reflectance were acquired to validate, respectively, the results of Module A and the AC correction performed by Module B. The airport of the city of Venice (45.4° N, 12.5° E, in the north-eastern part of Italy), shown in Figure 1, was selected as fulfilling the requirements mentioned above.

#### Airborne remote sensing data

The airborne sensor is the Daedalus AA5000 MIVIS (Multispectral Infrared and Visible Imaging Spectrometer), a whisk broom instrument, owned by the Italian National Research Council (CNR). Table 1 shows the characteristics of this airborne sensor.

The MIVIS sensor belongs to the first generation of hyperspectral imaging sensors as it is composed of 102 channels and characterized by a Full Width at Half Maximum (FWHM) between 8 and 20 nm [23–26]. In particular, the 20 channels with continuous sampling and a spectral resolution of 20 nm in the VNIR spectral domain are considered in this work.

Currently, the Airborne Laboratory for Environmental Research (LARA) Division of the Institute for Atmospheric Pollution (IIA) of the Italian National Research Council (CNR) works with the MIVIS data available from airborne campaigns performed in the framework of both international and national projects.

Five images of the Venice airport (acquired on July 26, 2001 and July 20, 2002) were available. In Table 2, the ancillary data of the flight for each image, are reported.

#### At-ground data

**Figure 2.**
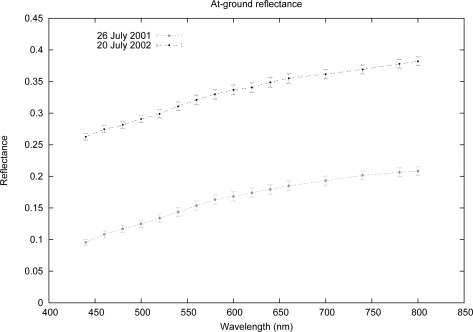
The reflectance of the surface, which meets the requirements of the isotropy of the BRDF and is spectrally homogeneous.

The surface reflectance was acquired using the FieldSpec Full-Range spectrometer [34]. The instrument characteristics are presented in Table 3. The hypothesis about the isotropy of the BRDF was confirmed by the at-ground measurements in the parking area at the Venice airport. The measurements, performed at different angles, ensure that the variability of the reflectance along the illumination and view angles is negligible (the BRDF obeys the theorem of reciprocity: interchanging the angles of incidence and reflection does not change the BRDF value). The *in situ* measurements of reflectance were collected to examine the spatial variability and the representative quality of the surface reflectance of the selected study area (*i.e.*, the parking area). Figure 2 shows the mean reflectance obtained from the measurements acquired on July 26, 2001, and July 20, 2002, with their standard deviations. The mean reflectance is the spectra used for the validation of the Module B results obtained from the available MIVIS images for 2001 (Images A, B and C) and 2002 (Images D and E).

The difference between the surface reflectances measured at the Venice airport in 2001 and 2002 could be caused by the natural progressive loss of pavement material from the asphalt surface due to weathering processes and induced corrosion mechanism. Furthermore, the measured reflectance could be affected by defects in the asphalt pavement due to a diminished petroleum content over time. For example, the reflectance measured in 2001 could have been lower than that measured in 2002 because wear on the asphalt revealed more limestone, which has a higher reflectance, underneath it. Conversely, a newly paved surface would lower the measured reflectance.

At the same time, measurements of *τ*_550_ were performed during the airborne overpasses by using the CIMEL sun-sky radiometer belonging to the AErosol RObotic NETwork (AERONET) federated international network [35], which has been located inside a 30 × 30*km*^2^ window where the atmospheric state is considered invariant for aerosol optical retrievals from remote sensing data, as explained in [21]. As explained in [36], the Venice site shows an increase of the aerosol optical thickness with weekly periodicities larger than the diurnal variability. These periodicities are a result of human activity that leads to a characteristic weekly emission cycle in an area of considerable size. The aerosol optical thickness is provided by the AERONET during the days of the campaigns (Thursday July 26, 2001 and Saturday July 20, 2002). On Thursday, July 26, 2001, a constant optical thickness during the whole period of available measurements from the Venice site (morning) could be assumed, whereas for Saturday, July 20, 2002, *τ*_550_ increased considerably during the day and became significant in the afternoon.

### 3.2. Retrieval of the Aerosol Optical Thickness, τ_550_ - Module A

The aerosol optical retrieval, *τ*_550_, and the surface reflectance of the study area, $\rho_{i}^{s}$, were assessed by the minimization of the cost function, Equation 3, between the at-sensor radiance measured by the MIVIS and the ones obtained from the Equation 1.

The MIVIS radiance selected for the minimization is within the atmospheric window of the VNIR spectral domain to drive the investigation of the effects of aerosol scattering neglecting the beam attenuation due to the absorption process: $L_{i}^{\mathrm{meas}}$ with *i* ∈ 440, ..., 800*nm* sampled every 20 nm. In this spectral domain, four channels were removed. The discarded values of $L_{i}^{\mathrm{meas}}$ are within the absorption bands of atmospheric constituents, the $L_{i=680\mathrm{nm}}^{\mathrm{meas}}$ and $L_{i=760\mathrm{nm}}^{\mathrm{meas}}$ of *O*_2_ as well as the $L_{i=720\mathrm{nm}}^{\mathrm{meas}}$ and $L_{i=820\mathrm{nm}}^{\mathrm{meas}}$ of *H*_2_*O* [37], as they are not useful for computing the columnar content of the atmospheric gas (MIVIS lacks the 940 nm channel which is sensitive for water vapor retrieval).

In addition to the aerosol optical thickness, the other atmospheric parameters used as input to radiative transfer calculations for simulating the radiative quantities of Equation 2, were fixed by the standard models included in 6S code. In particular, the mid-latitude summer atmosphere model was assumed for the meteorological parameters (pressure, temperature, water vapor and ozone) and the aerosol model, assumed in this work, was continental because in the Venice lagoon (close to an industrial site) the aerosol optical thickness is currently dominated by the fine fraction mode as explained in [36]. Afterwards, to simulate the $\rho_{i}^{\mathrm{sim}}$ in the atmospheric window following Equation 1, the retrieved radiative quantities of Equation 2 were convolved to the spectral response function of each MIVIS channel with a spectral sampling at 2.5*nm*.

The spatial resolution of the available MIVIS images is always less than 7 m and the number of pixels used in this method is from 2 to 5, which means that the surface area is ranging from 98*m*^2^ to 245*m*^2^. This amount is enough to neglect variability of the aerosol optical thickness inside the study area. From this approach, the aerosol optical thickness is constant inside a window with spatial resolution of 30 × 30 km, that satisfies the invariance of the atmospheric state for aerosol retrieval, as explained in [21]. On other hand, the number of pixels needed to apply the algorithm, depends on the spatial resolution of the airborne image. The surface area covered by the aircraft parking has been verified to be a homogeneous and Lambertian surface for much more than 245*m*^2^ thus the used pixels meet these requirements for the images with high spatial resolution: 2.3 m (image D) and 2.5 m (image E) and for the images with low spatial resolution 6.6 m (image A), 6.7 m (image B), 6 m (image C).

The minimum of the cost function (Equation 3) was reached for each MIVIS image, thus determining the values of the free parameters (*i.e.*, *τ*_550_ and $\rho_{i}^{s}$). *τ*_550_ is a sensitive parameter depicting the accuracy of the atmospheric radiative effects on the at-sensor radiance. The retrieved values of the aerosol optical thickness were validated with the at-ground measurements performed by the Cimel sun-sky radiometer.

The table 4 shows the CIMEL aerosol optical thickness available from the Venice AERONET station with the corresponding level of data quality, usually employed for aerosol studies [38,39]. The results in Table 4 are in agreement with the at-ground measurements with a good correlation index (*r*^2^ = 0.75) and low root mean square deviations (*RMSD* = 0.08), leading to the assumption that the altitude of the aerosol is in the boundary layer. In fact, a previous study confirmed the limitation of the method to retrieve aerosol properties above the aircraft, such as in the case of dust intrusion [5].

In Table 4, the high value of *τ*_550_ retrieved for Image D (Table 2) is probably attributable to the atmospheric aerosol above the aircraft altitude, which has been verified to limit the method’s performance in evaluating the optical parameter, [5]. Table 2 shows that Images A, B and C were acquired at higher altitudes than the Images D and E. Consequently, the results highlight the difficulty of using this method for retrieving the aerosol optical thickness when applied to data acquired at a low altitude.

The linear fit applied to the *τ*_550_ resulting from Module A versus the reference values measured at-ground, as represented by the *y* = *ax* straight line, returned a slope of *a* = 0.80.

### 3.3. Pixel-Based Atmospheric Correction of the MIVIS image - Module B

Module B of the method focuses on the pixel-based AC of the MIVIS image by using a physically-based algorithm to solve the direct problem, with the 6S simulation of the radiative quantities that takes advantage of the *τ*_550_ retrieved by Module A.

**Figure 3.**
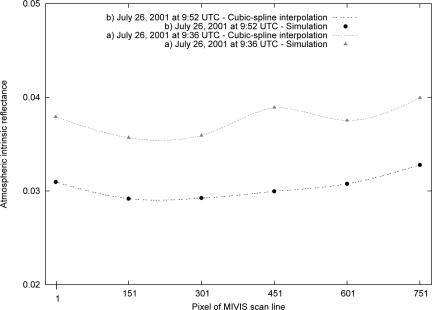
The path radiance *L^atm^*, as expressed in reflectance (namely, the atmospheric intrinsic reflectance *ρ^atm^*), simulated by the 6S code (circle and triangle) and interpolated by the cubic spline for a scan line of the MIVIS image acquired on July 26, 2001 at 9:36 UTC (grey) and at 9:56 UTC (black).

The retrieval of the final *ρ_i_* for each pixel of the MIVIS images was defined starting from the assessment of $\rho_{i}^{s}$ by using the analytical inversion of Equation 1. The simulation for modeling the radiative field to determine all of the radiative quantities ( $g_{i}^{j}$) of the equation was performed only in some pixels of a scan line, with a step of 150 pixels (the MIVIS scan line is composed of 755 pixel). The other $\rho_{i}^{s}$ of the image were retrieved by the cubic spline [30] applied to the radiative quantities simulated by the 6S code. Figure 3 shows two examples of *ρ^atm^* used to solve Equation 1 for all of the pixels of a scan line relative to the geometric conditions of Images A and B. The circles and triangles are the simulated values for six pixels of a scan line, and the discrete line segments are the values calculated for each pixel of the scan line by the cubic-spline interpolation. The radiative quantities ( $g_{i}^{j}$) of a scan line can be used for all of the scan lines of an image because the morphology of the scene is flat, ensuring the invariance of the radiative quantities.

Finally, the environmental contamination is removed by Equation 4, obtaining the final reflectance, *ρ* ∈ *ρ^i^*, where {*i* = 1, ..., 16} are the MIVIS channels. < $\rho_{i}^{s}$ > was calculated for each MIVIS channel in every pixel by the mean reflectance value of the surrounding pixels. In general, the pixel-based approach to removing the adjacency effect could increase the computational time; therefore, the encoded algorithm calculating the mean is the least-cost processing method of the remote sensing data for evaluating the environmental contribution.

**Figure 4.**
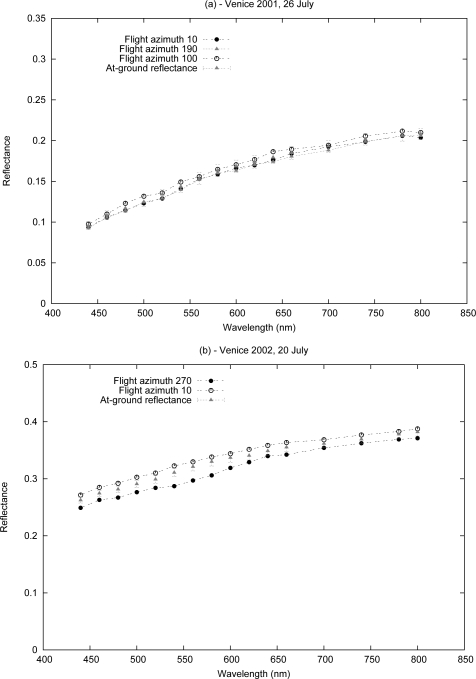
The surface reflectance, *ρ*, for all of the MIVIS channels, in the parking area of the Venice airport measured at-ground using the FieldSpec spectrometer (convolved from the MIVIS response function) and retrieved from the atmospheric correction (Module B) of the MIVIS images acquired on (a) July 26, 2001; (b) July 20, 2002. The scene was acquired by the MIVIS sensor at different geometric conditions: flight azimuth, date and time ensuring that the method was checked for different values of the angular variables: (*μ_s_*, *μ_υ_*, *ϕ*).

The validation of the *ρ_i_*, obtained from the atmospheric correction (Module B) of the MIVIS images acquired on July 26, 2001, and July 20, 2002, was performed on the selected study area (*i.e.*, the parking area at the Venice airport) with data acquired at-ground using the FieldSpec spectrometer. The spectral reflectance target of the study area is plotted in Figure 4 (a–b). The reference values for the reflectance measured at-ground and sampled every 1*nm*, were convolved to the MIVIS response functions, following Equation 2, to compare them with the results of this module. The scene is acquired by the MIVIS sensor at different geometric conditions defined by the flight azimuth, *ϕ_υ_*, the time of acquisition, *θ_s_*, and, consequently, the view zenith angle, *θ_υ_*.

**Figure 5.**
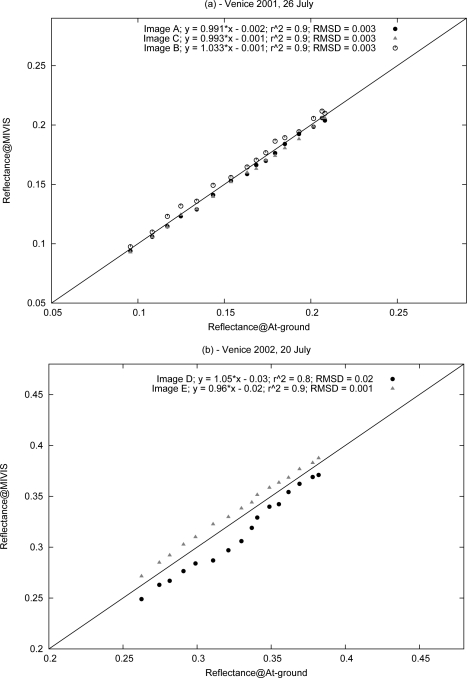
The linear fit of the study area’s measured reflectance (Reflectance@At-ground) and the reflectance retrieved from Module B for all of the MIVIS channels (Reflectance@MIVIS) for the image acquired on (a) July 26, 2001 and (b) July 20, 2002.

Figure 5 (a–b) represents the reflectance obtained from the MIVIS images versus the at-ground reflectance. The high correlation index (*r*^2^ = 0.9) and low value of the root mean square deviations (*RMSD* = 0.003) for the three MIVIS images of July 26, 2001 (A, B and C) are shown in Figure 5(a). The values of these statistical parameters attest to the reliability, in terms of the accuracy and repeatability, of the results obtained from Module B for all of the sensor channels belonging to the atmospheric window in the VNIR spectral domain.

Figure 5(b) shows that the surface reflectance, retrieved for Image D, is less comparable to the at-ground measurements than that obtained from other images (*r*^2^ = 0.8 and *RMSD* = 0.02). The cause, for Image D, could be attributed to the difference between the aerosol optical thickness obtained from the MIVIS data and the data obtained at-ground by the Cimel sun-sky radiometer (Table 4). The errors in the aerosol optical thickness retrieval led to an increase in the sizes of the errors of the direct and diffuse components of the transmittance simulated by the radiative transfer code and used in Equation 4. Consequently, the adjacency effect was not completely removed from the image. Therefore, the neighbor pixel reflectance was still present on the retrieved spectrum of the study area [40].

To evaluate the accuracy of the results, a linear regression between the results of Module B and the measured surface reflectance, was performed (Figure 5). The values of the key parameters, with a slope close to 1 and an intercept close to 0, confirm the quality of the results and highlight the repeatability of the presented method with different geometric conditions, as shown from the results for July 26, 2001, and July 20, 2002.

## 4. Conclusions

A new approach to aerosol optical retrieval from high spectral resolution remote sensing data and their atmospheric correction is presented. The method uses all of the radiative information inherent to the atmospheric window of the VNIR spectral domain and does not require any a priori knowledge of the values of the aerosol optical thickness and the spectral reflectance of reference pixels. The physically-based method is composed of Module A, which is devoted to the aerosol optical thickness (*τ*_550_) retrieval by solving the inverse problem, and Module B, which is used for the pixel-based atmospheric correction of the remote sensing data, by solving the direct problem. Both of the modules are versatile enough to be adapted to different geometric conditions and spectral characteristics of the sensor. The presented method was developed by separately considering the contributions of each sensor channel to the atmospheric radiative effects on the at-sensor signal for solving the inverse problem.

In the present work, the first application of the method to airborne data was presented using five MIVIS images and considering the channels on the VNIR spectral domain, where the aerosol contribution has much more weight compared to the other spectral domains. Module A allows the direct retrieval of the aerosol optical thickness (*τ*_550_) from the at-sensor signal without the use of ancillary data like aerosol optical thickness and surface reflectance measurements. In contrast, Module B retrieves the surface reflectance (*ρ*) of each pixel of the image for the sensor channels falling into the atmospheric window of the VNIR spectral domain.

The *τ*_550_ retrieved by Module A shows a clear correlation with the values measured at-ground using a sun-sky radiometer (*r*^2^ = 0.75 and *RMSD* = 0.08). The processing of the first image acquired on July 20, 2002 (Image D), leads to a *τ*_550_ value that is not comparable to the at-ground measurements, probably due to the presence of the aerosol above the aircraft, [5]. Further studies will be needed to analyze the radiative effects on the signals of airborne sensors due to aerosol above the aircraft.

The *ρ* retrieved by Module B, in comparison with the surface reflectance measured at-ground (high correlation index *r*^2^ ≥ 0.8 and low root mean square deviation *RMSD* ≤ 0.02) attests to the reliability of the atmospheric correction of the MIVIS data when acquired with different viewing geometries (view zenith and azimuth angles) and different times (solar zenith and azimuth angles).

Further studies will be focused on the applicability of the method to the angular directionality of the reflectance and to the channels falling into the absorption bands of the atmospheric constituents, which will be useful for columnar content retrieval. In fact, preliminary studies of the coupling of scattering and absorption and the BRDF model on the 6S code have already been performed to extend the method spectrally and are, without exception, based on the isotropy of the surface reflectance.

## Figures and Tables

**Table 1.** MIVIS sensor technical details and spectrometer characteristics.

Channels Number: 102		Instantaneous Field of View: 2.00*mrad*	
Spectral Coverage: 430 *−* 12700*nm*		Sample rate (angular step): 1.64*mrad*	
Pixels per scan line: 755		Total Scan Angle (FOV): 71.059°	

Spectrometer	Range (*nm*)	Channels (#)	bandwidth (*nm*)
I	430 − 830	20	20
II	1, 150 − 1, 550	8	50
III	2, 000 − 2, 500	64	8
IV	8, 200 − 12, 700	10	450

**Table 2.** The ancillary data for each MIVIS image.

Image	Date	Time (UTC)	Azimuth, *ϕ_υ_* (degrees)	Altitude, *z* (*km* a.s.l.)
A	July 26, 2001	9:36	10°	3.989
B	July 26, 2001	9:52	100°	4.051
C	July 26, 2001	10:01	190°	3.649
D	July 20, 2002	14:48	270°	1.406
E	July 20, 2002	15:22	10°	1.521

**Table 3.** The characteristics of the FieldSpec spectroradiometer.

Spectral range	350–2500 nm
Spectral resolution	3 nm@700 nm; 10 nm@1400/2100 nm
Spectral sampling	1.4 nm@350–1,050 nm; 2nm@1,000–2,500 nm
Noise Equivalent delta radiance (NeDL)	1.4 × 10^−9^*W/cm*^2^*/nm/sr*@700 nm 2.4 × 10^−9^*W/cm*^2^*/nm/sr*@1,400 nm 8.8 × 10^−9^*W/cm*^2^*/nm/sr*@2,100 nm

**Table 4.** *τ*_550_ measured by the CIMEL with the level of data quality available from the AERONET station and retrieved by the Module A for all of the MIVIS image available at the Venice image at the Venice airport.

Image	*τ*_550_@CIMEL	data quality	*τ*_550_@MIVIS

A	0.319	level 1.5	0.389
B	0.290	level 1.5	0.355
C	0.280	level 1.5	0.295
D	0.115	level 2.0	0.269
E	0.154	level 2.0	0.115
